# A new initiative to track HIV resource allocation and costs

**DOI:** 10.2471/BLT.22.287818

**Published:** 2022-06-01

**Authors:** Ryan McBain, AK Nandakumar, Michael Ruffner, Carlyn Mann, Mai Hijazi, Susanna Baker, Linden Morrison, Kalipso Chalkidou, Shufang Zhang, Iris Semini, Fern Terris-Prestholt, Steven Forsythe, Sarah Byakika, Joshua Musinguzi, Robert S Kaplan

**Affiliations:** a Partners in Health, 800 Boylston Street, Boston, Massachusetts 02199, United States of America (USA).; b Office of the Global AIDS Coordinator at the United States Department of State, Washington, DC, USA.; c United States Agency for International Development, Washington, DC, USA.; d The Global Fund to Fight AIDS, Tuberculosis and Malaria, Geneva, Switzerland.; e Fast Track Implementation Department, Joint United Nations Programme on HIV/AIDS, Geneva, Switzerland.; f Avenir Health, Glastonbury, USA.; g Ministry of Health, Kampala, Uganda.; h Harvard Business School, Boston, USA.

In early 2020, several global health institutions – including the Global Fund to Fight AIDS, Tuberculosis and Malaria, Joint United Nations Programme on HIV/AIDS (UNAIDS), United States Agency for International Development and Office of the Global AIDS Coordinator at the United States Department of State – aligned behind a new multicountry initiative to track resource allocation and funding for human immunodeficiency virus (HIV) services throughout sub-Saharan Africa.^1^^,^^2^ This initiative, known as activity-based costing and management, will generate detailed information, down to the patient level, on how we are tackling HIV.

The initiative recognizes that country governments and global institutions need to align and optimize their investments, particularly against the backdrop of an ongoing transition in which country governments are assuming incrementally greater fiscal responsibility for service delivery.^3^^,^^4^ The initiative also acknowledges the challenges inherent in this undertaking,^5^^,^^6^ and therefore seeks to provide detailed information on where current investments are being directed and how this transition is shaping programmes in reality.

As a global coalition, the initiative will empower institutions to gather patient-level information on HIV resource allocation and then share findings through a learning collaborative model. At the initiative’s centre is a unified approach to cost accounting known as time-driven activity-based costing.^7^^,^^8^ The principle of this approach is simple: measure the costs of all resources used to care for patients as they move through the health system, and use time as the measurement unit for assigning costs to resources. Modal pathways are estimated from the aggregate of observations across all patients

This approach has two advantages over alternative frameworks. First, it operates at the patient level by directly observing and measuring patients’ interactions and movements through the health system. Second, it reveals departures from protocols, along with the degree of variation in resources allocated to patients.^9^

The inclusion of this variance parameter plays an instrumental role in programme evaluation because it allows policy-makers to analyse patient-level information to determine what predicts why some patients receive more resources, while other patients receive fewer. 

The initiative is now underway in Kenya, Mozambique, Namibia, Uganda, United Republic of Tanzania and Zambia, with plans to expand beyond sub-Saharan Africa. In each setting, a local steering committee has determined the scope of operations, appointed local implementation partners to collect and analyse data and engaged in dissemination efforts with relevant ministries. Dissemination will include country-level reports and facility-level analyses to share with participating health clinics and hospitals. Over the medium to long term, a cross-country data analytics hub will work closely with steering committees and local and international implementation partners to produce academic publications and interactive dashboards that routinely monitor health system performance, based on information from the activity-based costing and management approach.

From a funding perspective, the initiative should allow participating institutions to assess how much of their investments actually reach health-care facilities, as well as quantify the impact of the financing transition on facility-level resources and out-of-pocket spending. The reach of the initiative across countries will also allow institutions to exercise their convening power to bring country leaders together to compare and assess alternative HIV care delivery models and inform future investments. 

The initiative also brings challenges. The method has been criticized for being technocratic, cumbersome and resource intensive.^10^ The initiative’s implementers have sought to mitigate these concerns by developing standardized curricula and data collection tools that support capacity-building. We have also developed template patient consent forms, institutional review board documentation and protocols to remove personal identifiers. Furthermore, while activity-based costing and management has been used in high-income countries primarily for cost reduction, its application in low-resource settings is likely to reveal situations where greater resources are desirable. Whether trimming or expanding resources, a main emphasis of the initiative should be to strengthen value for money and optimize programme performance.

Early findings from the initiative will be shared later this year. The President’s Emergency Plan for AIDS Relief and the Global Fund will also launch a Secretariat to coordinate the initiative’s activities across participating countries and begin the collaborative learning component. The initiative could also serve as a model for examining other health services.
